# Stem and Progenitor Cell Subsets Are Affected by JAK2 Signaling and Can Be Monitored by Flow Cytometry

**DOI:** 10.1371/journal.pone.0093643

**Published:** 2014-04-03

**Authors:** Ryuji Iida, Robert S. Welner, Wanke Zhao, José Alberola-lla, Kay L. Medina, Zhizhuang Joe Zhao, Paul W. Kincade

**Affiliations:** 1 Immunobiology and Cancer Program, Oklahoma Medical Research Foundation, Oklahoma City, Oklahoma, United States of America; 2 Beth Israel Deaconess Medical Center, Boston, Massachusetts, United States of America; 3 Department of Pathology, University of Oklahoma Health Sciences Center, Oklahoma City, Oklahoma, United States of America; 4 Department of Immunology, Mayo Clinic, Rochester, Minnesota, United States of America; B.C. Cancer Agency, Canada

## Abstract

Although extremely rare, hematopoietic stem cells (HSCs) are divisible into subsets that differ with respect to differentiation potential and cell surface marker expression. For example, we recently found that CD86^−^ CD150^+^ CD48^−^ HSCs have limited potential for lymphocyte production. This could be an important new tool for studying hematological abnormalities. Here, we analyzed HSC subsets with a series of stem cell markers in JAK2V617F transgenic (Tg) mice, where the mutation is sufficient to cause myeloproliferative neoplasia with lymphocyte deficiency. Total numbers of HSC were elevated 3 to 20 fold in bone marrow of JAK2V617F mice. Careful analysis suggested the accumulation involved multiple HSC subsets, but particularly those characterized as CD150^HI^ CD86^−^ CD18^L^°CD41^+^ and excluding Hoechst dye. Real-Time PCR analysis of their HSC revealed that the erythropoiesis associated gene transcripts Gata1, Klf1 and Epor were particularly high. Flow cytometry analyses based on two differentiation schemes for multipotent progenitors (MPP) also suggested alteration by JAK2 signals. The low CD86 on HSC and multipotent progenitors paralleled the large reductions we found in lymphoid progenitors, but the few that were produced functioned normally when sorted and placed in culture. Either of two HSC subsets conferred disease when transplanted. Thus, flow cytometry can be used to observe the influence of abnormal JAK2 signaling on stem and progenitor subsets. Markers that similarly distinguish categories of human HSCs might be very valuable for monitoring such conditions. They could also serve as indicators of HSC fitness and suitability for transplantation.

## Introduction

Hematopoietic stem cells (HSCs) normally replace blood cells according to need, but particular lineages are disproportionally expanded in myeloproliferative neoplasia (MPNs). The transition from normal, steady-state to disease likely involves HSC or very primitive hematopoietic progenitors, but it has been difficult to pinpoint such early changes [1], [2].

Though extremely rare within bone marrow, HSC are heterogeneous and divisible with recently developed methods [3], [4]. For example, single cell transplantation experiments revealed that some HSC preferentially generate myeloid or lymphoid lineage cells, while others are “balanced” with respect to blood cell formation [5]–[7]. New and less tedious barcoding approaches yielded essentially the same information [8], [9]. Importantly, only the myeloid-biased and balanced HSC have durable self-renewal properties. Individual HSC also differ with respect to time spent in a quiescent state and ability to produce blood cells for prolonged periods [10], [11]. At least some of these functional characteristics remain stable through multiple cycles of serial transplantation.

HSC subsets are also divisible on the basis of cell surface marker expression and fluorescent dye efflux [12]–[14]. For example, we found that unique CD150^Hi^ CD86^−^ HSCs from normal animals are poor at replenishing the adaptive immune system [15]. HSCs with those characteristics accumulate with age and in animals repeatedly exposed to small amounts of lipopolysaccharide [16].

The JAK2V617F mutation is found in more than 95% of polycythemia vera (PV) patients as well as in many others with MPNs [1]. The consequences of this abnormality have been extensively studied with experimental models where single or multiple copies of JAK2V617F were introduced to mice [17]–[28]. In all of these circumstances, JAK2V617F causes progressive erythro-megakaryocytic abnormalities, and myelofibrosis has occasionally been observed. Transplantation experiments suggest that abnormal JAK2 signaling affects HSCs in the lineage marker negative, Sca-1 antigen positive, c-Kit^+^ (LSK) fraction. In contrast, the disease is not transferrable by multipotent progenitor (MPP), megaryocyte-erythroid progenitor (MEP), common myeloid progenitor (CMP) and granulocyte-macrophage progenitor (GMP) subsets [23], [24], [29].

Much has been learned about intracellular signaling pathways that involve JAK2 and participate in disease. For example, total protein phosphorylation was increased in PV patients [30]. Also, deletion of Stat 1 and 5 in JAK2 knock-in mice blocked disease progression [31], [32]. The thrombopoietin receptor (TpoR or MPL) is known to be important for HSC self-renewal. Expression of mutant JAK2V617F in cultured cells reduced TpoR protein, inhibited apotosis and promoted cell division [33].

A potential therapy was suggested by the fact that IFNα selectively modulates the JAK2V617F burden in HSC in mice [2], [34]. However, pegylated interferon alpha-2 may have increased numbers of somatic mutations outside of the JAK-STAT pathway in a clinical trial [35]. JAK2 phosphorylates the arginine methyltransferase PRMT5, leading to increased genetic instability [36].

It should be possible to use HSC characteristics to monitor early changes in such hematologic diseases, and we have now tested that with a model system. We selected a JAK2V617F transgenic model, in part because there was previous evidence for lymphopenia [21]. Overgrowth of HSC predicted to have poor lymphoid potential correlated with reduced lymphocyte progenitor numbers, but not their ability to differentiate. If markers of equivalent utility can be found for human HSC, they might be valuable for predicting rebound of the immune system following chemotherapy and marrow transplantation.

## Results

### HSC Numbers are Increased in JAK2V617F Transgenic Mice

**Figure 1:**
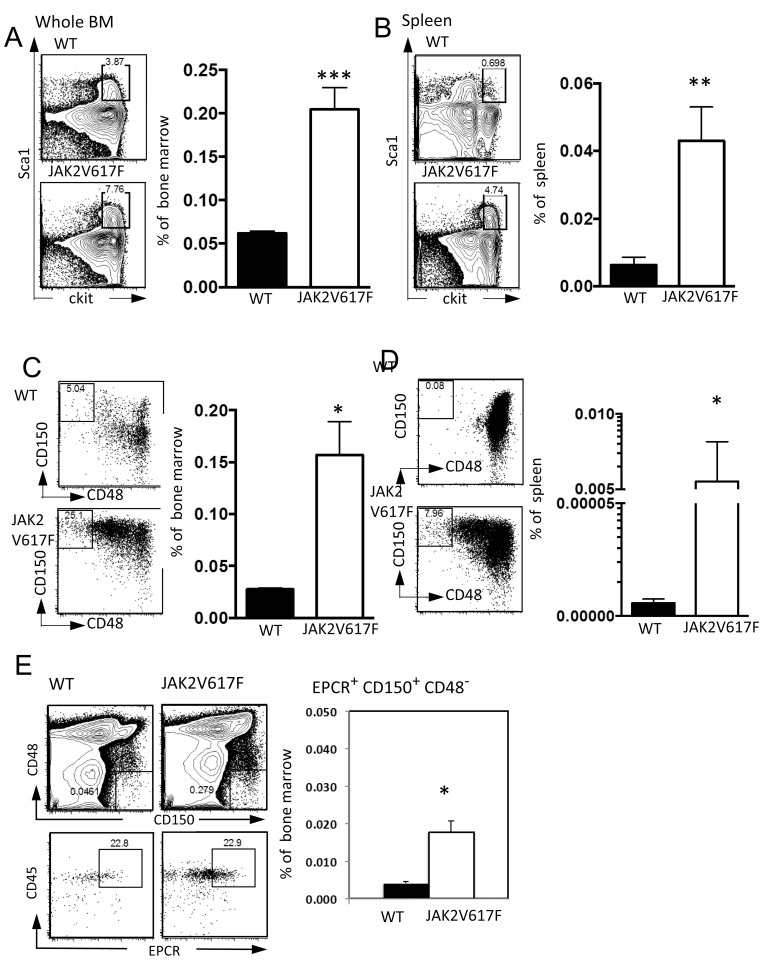
LSK and CD150^+^ CD48^−^ HSCs expand in JAK2V617F Tg mice. (**A, B**) LSK were gated with c-kit and Sca-1 in BM (**A**) and spleen (**B**). The percentages of LSK are shown(right panel in A and B). CD150^+^ CD48^−^ HSC in BM (**C**) and spleen (**D**) are given. Closed and open bars indicate WT and JAK2V617F individual mice, respectively. The data are representative of those obtained in two independent experiments (N = 8, 4) (**E**) Distinct HSC were resolved as EPCR^+^ CD45^+^ CD150^+^ and CD48^−^. The percentages of CD45^+^ EPCR^+^ CD150^+^ CD48^−^ in 10^6^ BM were increased in JAK2V617F mice. The data are representative of those obtained in two independent experiments (N = 6). *p*<0.001(***), *p*<0.01(**), *p*<0.05(*).

A variety of experimental strategies have been used to introduce single or multiple copies of the JAK2V617F mutation to mice [17]–[28]. Different levels and sites of expression in these models might account for different conclusions about the effects on HSC [37]. Although conditional knock-in mice have been described, PV patients can have multiple copies of the mutant JAK2V617F gene and we wished to exploit a robust model. Therefore, transgenic Line A animals [21] were studied between 10–21 weeks of age. Marrow cellularities were determined with two tibias from each mouse and significant differences were not found between any of the control and transgenic animals. However, cell numbers in the stem/progenitor rich Lin^−^ Sca-1^+^ c-Kit^Hi^ (LSK) fraction were significantly elevated (Fig. 1A). All of these animals had marked splenomegaly, and the incidences of LSK were also increased in that site (Fig. 1B). Elevations of more stringently gated CD150^+^ CD48^−^ within the LSK fraction [13] were also seen in both organs (Fig. 1C and 1D).

There are many phenotypic definitions of HSC. For example, Eaves and colleagues gate on EPCR^+^ CD45^+^ cells that are also CD150^+^ CD48^−^ [38]. There was a five-fold accumulation of HSC enumerated in this way in JAK2V617F mice (Fig. 1E). We conclude that the transgene expands numbers of HSC defined by two sets of inclusive criteria.

### A CD150^HI^ CD86^−^ CD18^L^°CD41^+^ HSC Subset Accumulates in JAK2V617F Transgenic Mice

**Figure 2:**
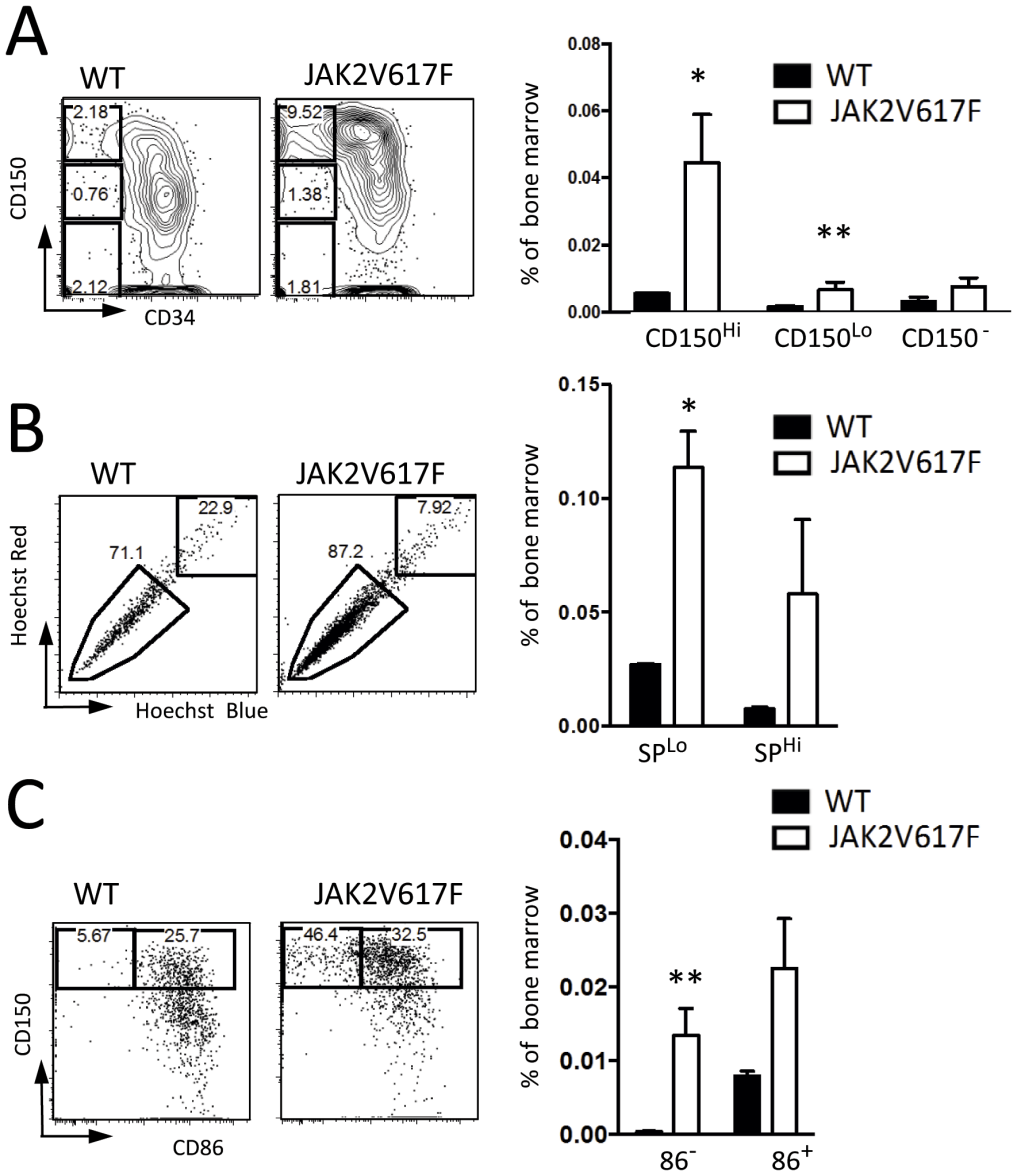
Particular HSC subsets expand in JAK2V617F Tg mice. (**A**) LSK were resolved with CD150 and CD34. CD34^−^ CD150^Hi^, ^Lo^, and negative HSC were gated as described by Morita [10]. The percentages of CD34^−^ HSC subsets in BM were calculated. Closed and open circles indicate individual WT and JAK2V617F mice, respectively. The data are representative of those obtained in two independent experiments (N = 6). (**B**) Lower side population cells in BM (spindle-shaped gating) are thought to include myeloid biased HSC. The “side population tip” was increased in JAK2V617F mice. Representative plots are shown in the left panels, while cell percentages of lower and upper cells are given in right panels. (**C**) Both CD86^−^ and CD86^+^ CD150^+^ CD48^−^ LSK cells in BM were increased in JAK2V617F mice. CD48^−^ LSK were resolved with CD86 and CD150. Cell percentages of CD86^−^ and CD86^+^ in CD150^Hi^ were calculated. The data are representative of those obtained in three independent experiments (N = 8) *p*<0.01(**), *p*<0.05(*).

Previous studies revealed that HSC are heterogeneous and composed of functionally specialized subpopulations [3], [4]. In some cases, this corresponds to phenotypes [10], [39], and the JAK2V617F transgene preferentially expands CD150^Hi^ HSC (Fig. 2A). That category of HSC was reportedly less likely to generate lymphocytes than CD150^Lo/−^ HSC [10]. Similarly, Goodell and colleagues found lymphopoietic potential was lowest among HSC that strongly exclude Hoechst dye [40]. These “lower side population” HSC were the most increased by JAK2V617F (Fig. 2B).

We recently found that a distinct population of CD150^Hi^ CD86^−^ HSC have poor lymphopoietic potential and accumulate with age or chronic Toll-like receptor (TLR) stimulation [15], [16]. Cells with this distinctive phenotype were prominent in the JAK2V617F transgenic mice (Fig. 2C). Percentages and numbers of LSK CD48^−^ CD150^Hi^ CD86^−^ HSC increased in response to JAK2 signaling in all of the transgenics (Fig. 2C). While most of the increases in this transgenic model involved CD86^−^ HSC, numbers of HSC expressing CD86 also increased in some of the animals.

CD18 staining patterns in healthy mice are similar to those obtained with CD86 [16], and that was also the case for transgenic mice (compare Fig. S1A with Fig. 2C above). In addition, we reported that CD41 expression on HSC increases with chronological age [15]. Another group concluded that CD41 acquisition corresponds to loss of lymphopoietic potential [41]. A similar phenomenon resulted from the JAK2V617F transgene in young mice (Fig. S1B). Although gating for CD41^−^ cells has been used as a strategy to enrich HSC [13], [41], CD41 was not included in our lineage depletion cocktail for all analyses. Interestingly, expression of CD41 tended to be reciprocal with CD86 on CD150^Hi^ CD48^−^ HSC (Fig. S1C). In contrast to these relationships, significant shifts were not recorded in HSC bearing VCAM-1 or CD39 (Fig. S1D, E). These markers were investigated because they appeared to change in a previous study involving TLR stimulation [15].

**Figure 3:**
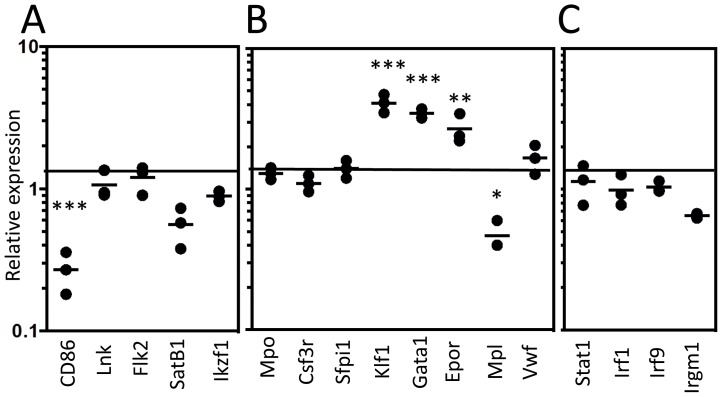
Erythropoiesis associated transcripts are overrepresented in JAK2V167F HSC. Gene expression analysis was performed by Real-Time PCR on a small panel of genes, normalizing with GAPDH for a reference housekeeping gene. Lymphoid genes (**A**)**,** myeloid-, erythroid-, and megakaryocytic- genes (**B**)**,** and IFNγ responsive genes (**C**) were indicated. The data are representative of those obtained in two independent experiments *p*<0.001(***), *p*<0.05(*).

We then sorted CD150^+^ CD48^−^ LSK cells from normal and JAK2V617F transgenic marrow for Real Time-PCR analyses with a small set of genes. This revealed depressed CD86 and Mpl transcripts, offset by increased expression of Klf1, Gata1 and Epor (Fig. 3A and B). In contrast, expression of Lnk, Flk2, Satb1, Ikzf1 HSC/lymphoid genes was unaltered (Fig. 3A). Stability of Mpo, Csf3r, Sfpi1 and Vwf lineage associated genes was also seen (Fig. 3B). The same PCR analyses were simultaneously performed and validated with sorted progenitors from normal mice (Fig. S2A and B). These are valuable when compared to Fig. 3B in showing that the transgene preferentially primes transcripts corresponding to primitive erythroid (CFU-E) lineage cells.

It seemed possible that the JAK2V617F transgene caused interferon pathway signaling as is the case in patients with essential thrombocythemia [30]. Also, HSC themselves are capable of Ifnγ production [42]. Stem/progenitors that normally lack Sca-1 could have acquired it as a result of such cytokine influence [43], [44]. However, Real-Time PCR analyses suggested that mechanism does not account for the expansion of LSK (Fig. 3C). That is, transcripts corresponding to Ifn inducible genes were not elevated.

Thus, the CD86^+^ HSCs that predominate in healthy young animals are present and occasionally even elevated in marrow of JAK2V617F transgenic mice. However, they are diluted by large numbers of Lin^−^ Sca-1^+^ c-Kit^+^ CD150^Hi^ CD34^−^ CD48^−^ CD86^−^ CD18^−^ CD41^+^ and/or Hoechst dye excluding cells. Earlier studies associated those properties with overlapping populations of HSC that are poor at replenishing the adaptive immune system [13]–[15]. Possibly related to that, JAK2 transgenic HSC had elevated expression of two transcription factors required for erythropoiesis.

### Primitive Hematopoietic Progenitors are Affected by JAK2 Signaling

**Figure 4:**
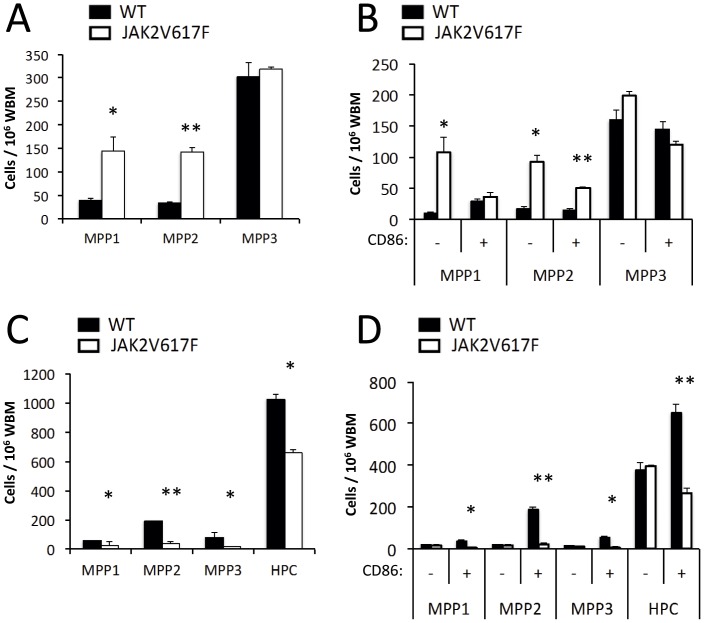
Multipotent progenitors are altered in JAK2V617F Tg mice. (**A**) LSK from control and Tg mice were resolved with CD150, CD48, Flk2 and CD34 staining into MPP subsets as described by Trumpp and colleagues [45]. The subset definitions are CD150^+^ CD48^−^ CD34^+^ LSK (MPP1), CD150^+^ CD48^+^ CD34^+^ LSK (MPP2) and CD150^−^ CD48^+^ CD34^+^ LSK (MPP3). (**B**) These populations were further subdivided on the basis of CD86 expression. (**C**) Morrison and colleagues exploited CD150, CD48, CD229 and CD244 to define MPP subsets [46], and (**D**) again we subdivided them on the basis of CD86 display. The surface markers define CD150^−^ CD48^−^ CD229^−^ CD244^−^ (MPP1), CD150^−^ CD48^−^ CD229^+^ CD244^−^ (MPP2), CD150^−^ CD48^−^ CD229^+^ CD244^+^ (MPP3), and CD150^−^ CD48^+^ (HPC). The results are given as the percentages of BM cells and are representative of those obtained in two independent experiments (N = 3). Despite the common nomenclature used with these methods, distinct subsets and possibly separate differentiation pathways are identified (See Figure 8).

Multipotent progenitors with limited self-renewal capability represent the immediate progeny of HSC and subsets of them have been defined with various collections of markers. A method described by Trumpp and colleagues [45] revealed that expansion of CD150^+^ CD48^−^ CD34^+^ LSK (MPP1) as well as CD150^+^ CD48^+^ CD34^+^ LSK (MPP2) but not CD150^−^ CD48^+^ CD34^+^ LSK (MPP3) occurs in our transgenic mice (Figure 4A). Further resolution of those subsets showed that most of this expansion involved MPP lacking the CD86 indicator of lymphopoietic potential (Figure 4B).

Morrison and colleagues recently used a different collection of markers to define a different series of multipotent progenitors, while retaining the same MPP nomenclature [46]. We found that this approach showed significant depletion of CD150^−^ CD48^−^ CD229^−^ CD244^−^ (MPP1), CD150^−^ CD48^−^ CD229^+^ CD244^−^ (MPP2), CD150^−^ CD48^−^ CD229^+^ CD244^+^ (MPP3), and CD150^−^ CD48^+^ (HPC) in the JAK2 mice (Figure 4C). Remarkably, this depletion primarily involved the lymphopoietic CD86^+^ progenitors (Figure 4D).

These changes suggest that multipotent cells capable of generating lymphocytes are either greatly diluted (analysis with the Trumpp marker scheme) or depleted (Morrison method) as a result of JAK2 signaling. Furthermore, they are consistent with those described above for HSC and again suggest that potential to replenish the immune system is probably diminished.

### Lymphoid Progenitors Progressively Decline in JAK2V617F Transgenic Mice

**Figure 5:**
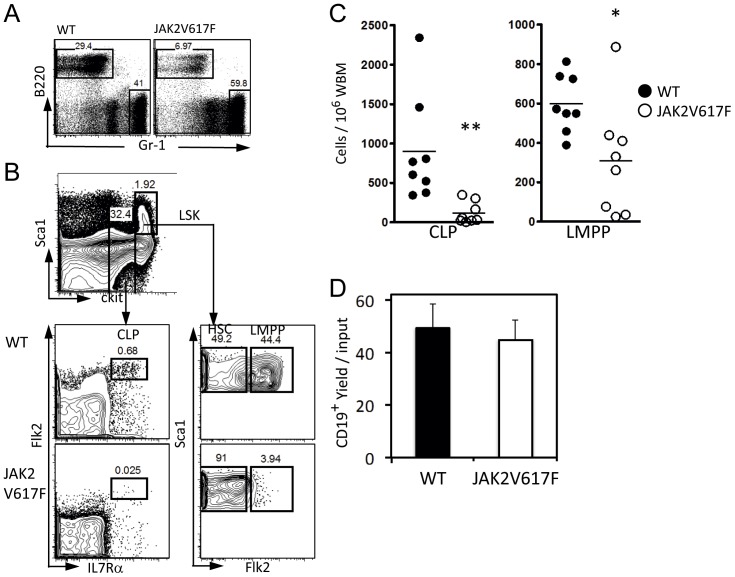
Lymphoid progenitors are depleted in JAK2V617F transgenic mice. (**A**) Whole bone marrow cells were characterized by staining with B220 (lymphoid specific) and Gr1 (myeloid specific) antibodies. (**B**) The c-kit^L^°CLP fraction was defined by staining for Flk2 and IL-7Rα (left panels). The LSK fraction was divided for enumeration of Flk2^Hi^ LMPP. (**C**) Percentages of CLP (left panel) and LMPP (right panel) are shown. The data are representative of those obtained in three independent experiments (N = 8). (**D**) Lymphoid potential is similar between WT and JAK2V617F CLP. CLP were cultured in serum- and stromal cell-free culture for 7 days. Production of differentiated CD19^+^ cells were calculated as yields per input progenitor. The data are representative of those obtained in two independent experiments *p*<0.01(**), *p*<0.05(*).

A previous analysis of this line of transgenic mice revealed small elevations in peripheral and bone marrow leukocytes, most likely granulocytes, as well as subtle changes in CD45R/B220^+^ lymphocytes [21]. We conducted a more thorough analysis of bone marrow and found evidence for reduced lymphopoiesis. That is, there were significantly reduced numbers of lymphoid lineage cells marked by B220 or CD19 expression coincident with expansion of Gr-1 bearing myeloid cells (Fig. 5A). As noted above, total marrow cellularities were unaffected by the transgene. Lineage marker negative (Lin^−^) subsets were then gated for analysis of primitive hematopoietic cells (Fig. 5B). That includes Lin^−^ c-Kit^Lo^ Flk-2^Hi^ IL-7Rα^+^ common lymphoid progenitors (CLP) that we found were depleted more than 10 fold in the transgenics (Fig. 5C, left panel). CLP are thought to derive from a fraction of Lin^−^ Sca-1^+^ c-Kit^Hi^ Flk2^Hi^ lymphoid primed multipotent progenitors (LMPP) [47], [48]. Numbers of these primitive lymphopoietic cells were also reduced (Fig. 5C, right panel). These changes were more pronounced in older transgenic animals (data not shown).

Given that the transgene is widely expressed in hematopoietic tissue, it was possible that lymphoid progenitors were directly affected. Therefore, we sorted the small numbers of CLP present in young JAK2V617F animals and placed them in stromal cell-free cultures that support B lymphopoiesis (Fig. 5D). Progenitors enriched in this way generated normal numbers of B lineage lymphocytes marked by CD19 expression.

We conclude that the JAK2 transgene affects early events in lymphopoiesis, depressing numbers of lymphoid progenitors. This is consistent with the erythroid associated gene expression in their HSC shown above. However, artificial JAK2 signaling in these animals did not interfere with the ability of residual lymphoid progenitors to differentiate. Those that escaped a JAK2 regulated checkpoint were fully able to generate B lineage lymphocytes.

### JAK2 May Slightly Influence Stem/Progenitor Cell Proliferation

**Figure 6:**
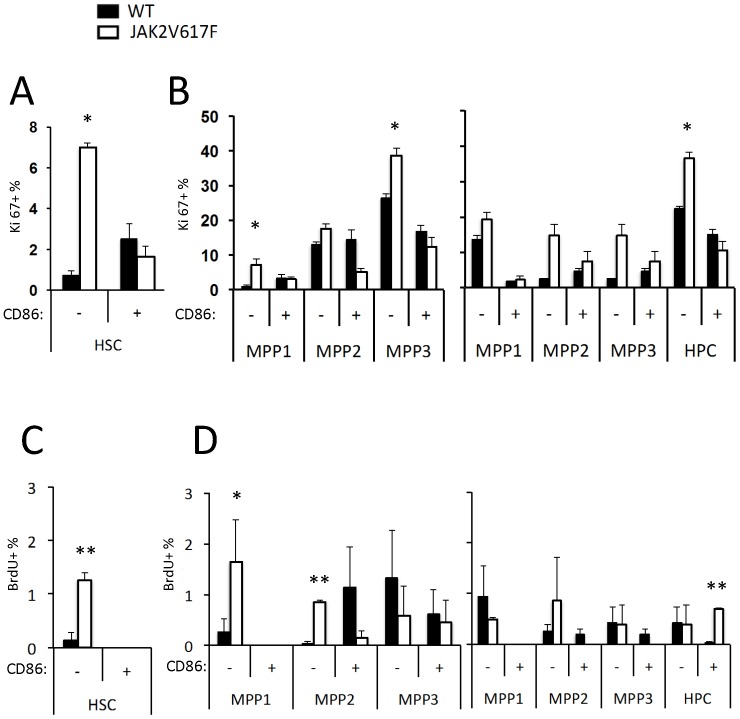
The JAK2V617F transgene may slightly alter proliferation of stem/progenitor cells. The CD150^+^ CD48^−^ HSC (**A**) and the multipotent progenitors (**B**) described Fig. 4 were stained with the proliferation associated Ki67 marker. Percentages of Ki67^+^ cells in BM were calculated. As another approach, the same marrow samples were pulsed with BrdU in very short-term cultures before staining (**C, D**)**.** These results are given as percentages of BrdU^+^ cells per subset and significance is indicated by asterisks *p*<0.01(**), *p*<0.05(*). These data were obtained in a single experiment (N = 3).

Increased self-renewal, prolonged survival and/or decreased export of stem/progenitor cells from the marrow could all account for their expansion in the transgenic mice. One of these parameters was assessed by staining HSC subsets with the proliferation associated Ki67 marker together with Hoechst dye (Figure 6A). While the results would be consistent with expansion of CD86^-^ HSC in JAK2 transgenics, they did not reach statistical significance and the same was true for some multipotent progenitor subsets (Figure 6B, Trumpp method, middle panel; Morrison method, far right panel). As another approach, BrdU was added to very short term (1 hr) cultures of bone marrow cells (Figure 6C, D). This revealed that there were significantly more dividing stem and progenitor cells in transgenic marrow. Note that all of these data were calculated and expressed in terms of percentages, but would reflect absolute numbers per bone because cellularities were unaffected. Even a small change in proliferation may be significant over time and it is interesting that the changes preferentially occurred in CD86^−^ cells.

### Two HSC Subsets can Transfer Myeloproliferative Disease

**Figure 7:**
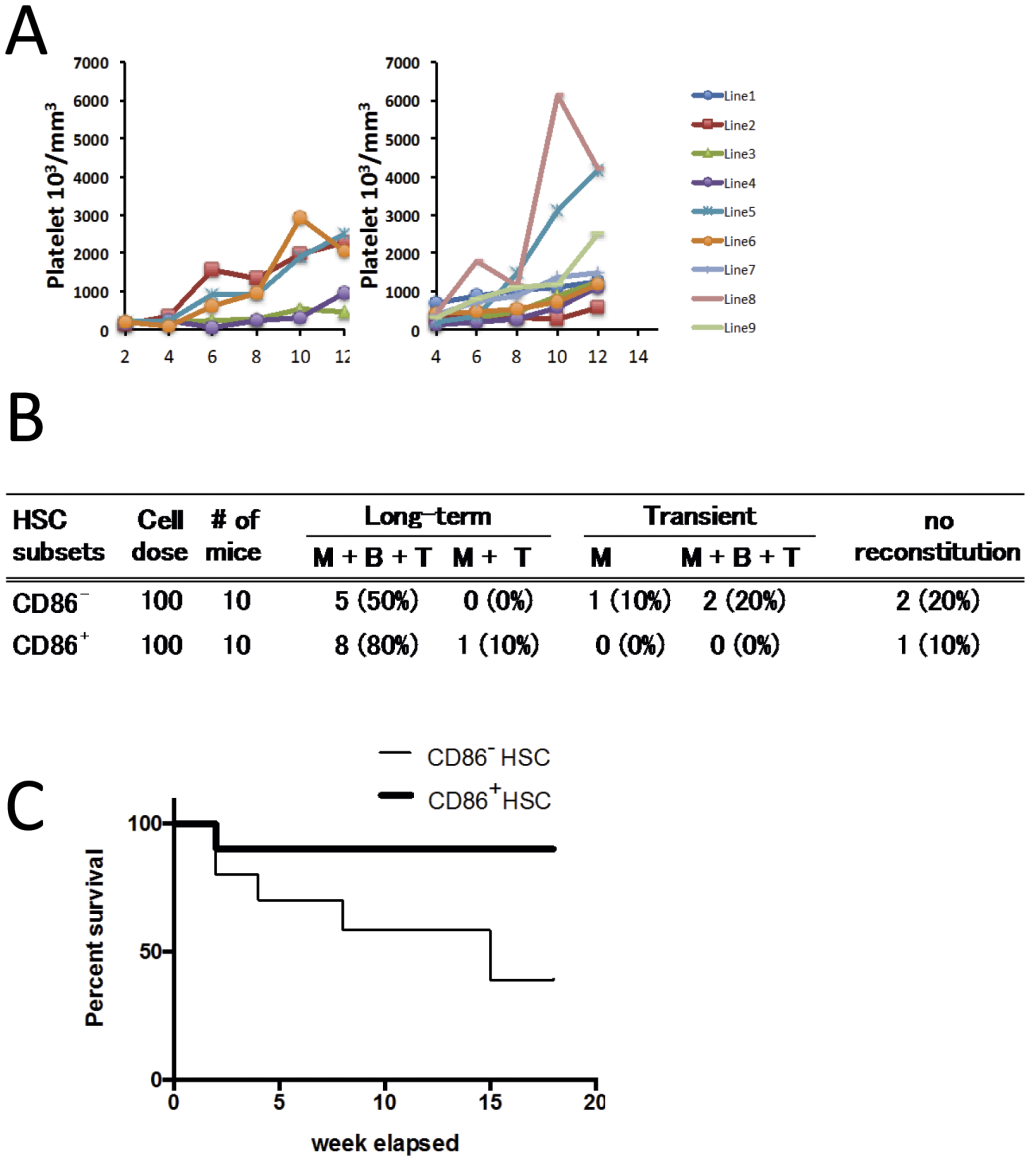
The JAK2V617F transgene initiates disease in both CD86^+^ and CD86^−^ HSC subsets. One hundred CD86^+^ or CD86^−^ CD150^+^ CD48^−^ HSC from CD45.2 JAK2V617F transgenic mice were transplanted to lethally irradiated CD45.1 recipient mice. (**A**) This table summarizes recipients that survived for at least 18 weeks after transplantation. Effective chimerism was considered if the recipients had more than 1.0% donor cells in the peripheral blood. (**B**) Platelet counts are shown at the indicated intervals. (**C**) Survival curves are given for recipients of the two HSC subsets.

Previous transplantation studies revealed that HSC, but not hematopoietic progenitors from JAK2V617F mice can transmit disease [2], [23]–[25], [29]. Therefore, we could now ask if normal versus disease differentiation decisions occur in a particular HSC subset. Marrow was harvested from 15 week old JAK2V617F animals that had high platelet (>4×10^6^/mm^3^) and RBC (>11×10^6^/mm^3^) counts as well as splenomegaly (>7 fold increased weight). The CD150^+^ CD48^−^ LSK cells were then sorted according to CD86 expression (as illustrated in Figure 2C) and 100 of each HSC subset were mixed with 10^5^ whole rescue marrow cells before transplantation into lethally irradiated mice (Figure 7). The design was such that progeny of CD45.2 marked transgenic HSC could be distinguished from rescue and recipient mouse cells identified as CD45.1.

Sampling of peripheral blood began 4 weeks later and continued for an additional 14 weeks. Engraftment was considered successful when >1.0% of total nucleated cells were persistently of donor type (Figure 7A). This occurred in five of ten CD86^−^ and nine of ten CD86^+^ HSC transplants. Animals were considered to have myeloproliferative disease when platelet counts exceeded 10^3^ per mm^3^ of blood. This occurred by 12 weeks post-transplant in three of the five (60%) CD86^−^ recipients as compared to eight of the nine (89%) animals that received CD86^+^ HSC (Figure 7B). Death tended to occur earlier in mice transplanted with the CD86^−^ HSC, and especially those with low chimerism (Figure 7C and data not shown). As described above, CD86^−^ HSC preferentially expand in response to JAK2 signaling (Figure 3C), but these transplantation results indicate that disease can initiate in either subset.

## Discussion

Many questions arise from recent findings relating to HSC heterogeneity. For example, are the properties of HSC populations affected by malignancies? At least some functional characteristics, such as the ability to restore the immune system, correlate with patterns of cell surface marker expression. That technical breakthrough suggested that flow cytometry rather than lengthy transplantation assays could be used to address some of these issues. Here we have tracked HSC subset changes when hematopoiesis was perturbed in a model of myeloproferative neoplasia. Dilution or depletion of HSC predicted to be lymphopoietic correlated with declines in common lymphoid progenitors.

Our prior studies indicated that loss of CD86 and CD18 together with increases in CD41 and CD150 defines HSC that accumulate in aged or chronically LPS treated animals [15], [16]. Even in untreated adult mice, such HSC are poor with respect to lymphocyte formation and probably overlap with myeloid-skewed HSC identified in single cell transplantation experiments [5]–[7]. While the JAK2V617F transgene caused accumulation of HSC defined with five commonly used methods, closer examination revealed that it primarily involved those that lacked CD86. In fact, most of them were Lin^−^ Sca-1^+^ c-Kit^+^ CD150^Hi^ CD34^−^ CD48^−^ CD86^−^ CD18^−^ CD41^+^ and/or Hoechst dye excluding cells.

**Figure 8:**
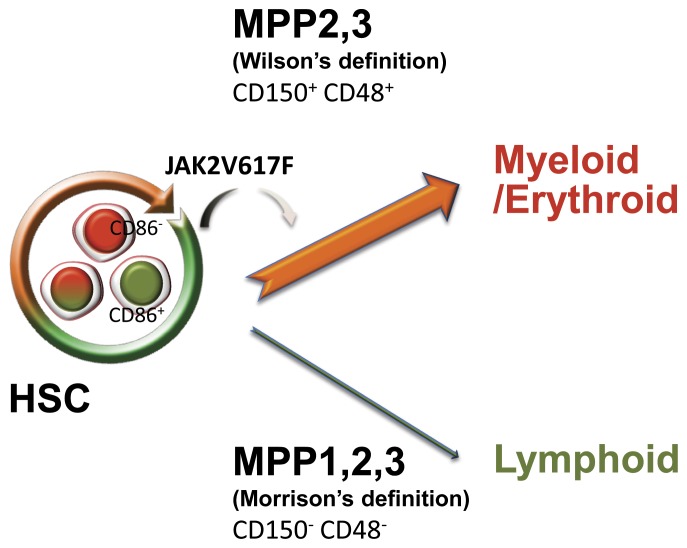
Two methods for resolving multipotent progenitor cells may identify alternate differentiation options for HSC. Trumpp and colleagues described a progression marked by acquisition of CD48 (MPP2 and 3) [45] and we show it as the top pathway. This option and particularly MPP that lack CD86, is promoted by the JAK2 mutant transgene. Alternatively, the Morrison lab showed that HSC can lose CD150 (formation of MPP 1, 2, 3 in the lower pathway) [46]. This route is diminished in the transgenics and most of the loss is in CD86^+^ MPP.

Multipotent progenitors with reduced self-renewal potential have been defined with various markers. Here we exploited two approaches that utilize the same nomenclature but describe different subsets of cells [45], [46]. The JAK2 driven changes in these subsets suggest alternative differentiation pathways may exist for these very primitive cells (Figure 8). Acquisition of CD48 marks one option (MPP1 to 2, 3 in the Trumpp scheme) and it is the one promoted by JAK2 signaling. Alternatively, HSC lose CD150 (formation of MPP 1, 2, 3 in the Morrison pathway), and this route is diminished in the transgenics. It is important to stress that the changes in MPP paralleled those in HSC and mainly involved CD86^−^ subsets. Further analysis of these distinct MPP populations might reveal if there are also differences in lymphoid versus myeloerythroid lineage priming.

We do not have a precise explanation for why particular populations expand and others contract in this model. Slightly increased proliferation in cells that are normally quiescent could be significant over time. It is noteworthy that JAK2V617F effects on stem cell proliferation are highly dependent on the model system used and need not parallel changes in progenitors [37].

It is interesting to compare manipulations of JAK2V617F to those involving Lnk. The latter is an adaptor molecule that negatively regulates JAK2 signaling via its SH2 domain [49]. That in turn inhibits downstream signaling mediated by Epor and MPL. Lnk deficient mice have increased numbers of HSC with enhanced self-renewal potential and increased quiescence [50]. Lnk^−/−^ mice also develop myeloproliferative neoplasia with thrombocytosis, splenomegaly and fibrosis [51]. The MPN phenotype develops more rapidly in Lnk^−/−^ mice that also have the JAK2V617F mutation [52]. Unlike the case with JAK2V617F transgenics, Lnk^−/−^ mice are characterized by B cell overproduction. Perhaps the impact of JAK2 pathway signaling in lymphopoietic cells is different in these two circumstances. Lnk can also inhibit c-kit and Flk2 signaling, raising the possibility that it influences lymphopoiesis independently of JAK2. The few CLP recovered from JAK2V617F transgenics retain normal potential to generate lymphocytes in culture, indicating they have no signaling abnormalities. Lnk expression was increased in CD34^+^ peripheral blood cells in MPN and PV patients with the JAK2V617 mutation [53], [54]. Although JAK2 and Lnk could be mutually regulated, we did not observe altered expression of Lnk in CD150^+^ CD48^−^ HSC from our Tg mice. Thus, our results indicate that the Lnk gene is not a direct or secondary target of JAK2V617F. Strong expression of JAK2V617F did reduce transcripts in HSC for Mpl, a Tpo receptor known to be important for hematopoiesis [55]. Also, JAK2 partially activates normal MPL function in HSC [56].

Results from our limited PCR analyses might reflect a shift in gene expression in HSC towards erythropoiesis, consistent with reduced numbers of LMPP and CLP. Lymphopoiesis might be further suppressed because potent HSC are diluted in lymphopoietic niches by CD86^−^ HSC. Regardless of the reason, our results are best explained in terms of an early JAK2 modulated checkpoint. Progenitors that progress beyond that point seem fully competent to generate lymphocytes.

As concluded by others, JAK2 affects HSCs, but JAK2V617F bearing ones can transfer the disease and in some models have a growth advantage over wild-type HSC [2], [29]. We now show that it preferentially involves a normally rare subset and speculate that JAK2 signaling can affect lineage choice decisions prior to the diversion of myelo-erythroid-megakaryocytic and lymphoid pathways. It is unclear if these CD86^−^ HSCs should be considered injured or re-programmed. Their staining characteristics predicted impaired lymphopoietic potential, and we found reduced numbers of functionally competent lymphoid progenitors. This could suggest that the immune systems of patients with JAK2 mutations are not being replenished to a normal degree.

These findings provide proof of principal that cell surface markers can be used to track HSC subsets in normal and disease circumstances. For example, we can now appreciate that selective expansion and mobilization of certain HSC occurs in MPNs. It is clear that more effort should be expended to identify and sub-divide HSC in humans.

## Materials and Methods

### Mice

C57BL/6 (Jackson Laboratory, Bar Harbor, ME) and JAK2 line A transgenics backcrossed for at least nine generations to C57BL/6 [21] were bred and maintained in the Laboratory Animal Resource Center at the Oklahoma Medical Research Foundation (Oklahoma City, OK) or the University of Oklahoma Health Sciences Center (Oklahoma City, OK). They were analyzed between 10 and 21 weeks of age. Experiments were performed in accordance with approved IACUC protocols.

### Ethics Statement

C57BL/6 (Jackson Laboratory, Bar Harbor, ME) and JAK2 transgenics backcrossed for at least nine generations to C57BL/6 [21] were bred and maintained in the Laboratory Animal Resource Center at the Oklahoma Medical Research Foundation (OMRF) (Oklahoma City, OK) or the University of Oklahoma Health Sciences Center (OUHSC) (Oklahoma City, OK). All experimental procedures were conducted under Institutional Animal Care and Use Committees. OMRF: Protocol KA-1251-1 (co-PI: Kincade and Alberola-Ila), approved November 15, 2012, and annually thereafter, Animal Welfare Assurance #A3127-01. OUHSC: Protocol #: 12-109-T (PI: Zhao), approved August 16, 2013, and annually thereafter, Animal Welfare Assurance #A3165-01.

### Antibodies and Flow Cytometry

Tissue and cell manipulations were performed in PBS with 3% fetal calf serum (v/v). Erythrocytes were lysed in NH_4_Cl^–^ hypotonic solution. Bone marrow was stained for 15 min on ice. Antibodies included APC-Cy7- lineage (CD3, CD8, CD11b, TER-119, NK1.1, CD19); FITC- FcgRIII(2.4G2), CD41(MWReg30); PE- CD86 (GL1); PE-Cy5- CD135/Flt-3 (A2F10); PerCP Cy5.5- CD48(HM48-1); PE-Cy7- CD150 (TC15-12F12.2); APC- EPCR (ebio1560), IL-7Rα (A7R34), Alexa645- CD34 (RAM34); Alexa700- Gr-1(RB6-8C5); Pacific Blue- Sca-1 (D7); Brilliant violet 510™- cKit (2B8); Brilliant violet 605™-B220 (RA3-6B2) and biotin- CD105 (MJ7/18). A secondary streptavidin PE-Texas Red was used for IL-7Rα staining Dead cells were excluded by fixable viability dye eFluorR780 (ebioscience). Cells were sorted using either a MoFlo (DakoCytomation, Ft. Collins, CO) or FACS-Aria cytometer (BD Biosciences, San Diego, CA). Purification of each subset was confirmed by post-sort analysis. For side population analysis, 10^7^ bone marrow cells were incubated with 5 μg/mL Hoechst 33342 in DMEM for 90 min at 37°C. Flow cytometry was performed on a BD LSRII (BD Biosciences, San Jose, CA), and FlowJo software (Treestar, San Carlos, CA) was used for data analysis.

### Cell Cycle Analysis

Cell were fixed and permeablilized with BD Cytofix/Cytoperm™ Fixation/Permeabilization solution after cell surface staining. Ki-67 was performed using the PE-mouse anti-human Ki-67 kit (BD Bioscience). For analysis of BrdU incorporation, bone marrow cells were incubated with 10 μM BrdU (Sigma) at 37°C for an hour. After cell surface staining, BrdU staining were performed using BD APC flow kit.

### Cell Culture

Sorted cells were cultured in round-bottom 96-well plates (Corning, Inc.) with X-VIVO15 medium (Biowhittaker, Walkersville, MD) containing 1% detoxified bovine serum albumin (Stem Cell Technologies, Vancouver, Canada), 5×10^−5^ M 2-mercaptoethanol (2-ME), 2 mM L-glutamine, 100 U/ml penicillin, and 100 mg/ml streptomycin. Culture medium was enriched with 100 ng/mL FL, 20 ng/mL SCF and 1 ng/mL IL-7. Incubation was maintained at 37°C in a 5% CO_2_ humidified atmosphere. Cells were fed by replacing half culture volume with fresh media and cytokines every three to four days. Cells were harvested at designated times and stained with monoclonal antibodies to CD19, B220, CD11c, Ly6c, CD11b/Mac-1, and NK1.1.

### Transplantation

Recipient (CD45.1) mice were lethally irradiated (2×6.5 Gy [650 rad]) with a 137Cs source (Mark I irradiator; J. L. Shepard and Associates). Mice were anesthetized with isoflurane, and cells were infused intravenously by retro-orbital injection. For purified HSC transplantations, HSCs were sorted directly into 96-well plates containing rescue marrow cells (1×10^5^ cells/200 μL). Competitive repopulation was assessed at 2-week intervals by peripheral blood analysis.

### Real-Time PCR

The mRNAs were isolated from sorted cells with Trizol (Invitrogen). The cDNAs were then prepared by using random primers and Moloney murine leukemia virus reverse transcriptase (Invitrogen). Reactions were quantified with fluorescent TaqMan technology. TaqMan primers and probes specific for every genes were used in the ABI7500 sequence detection system (Applied Biosystems) using QuanTitect PCR Mix (Qiagen). Reactions were run at an annealing temperature of 60°C with 45 cycles. Each sample was measured in triplicate, and the comparative threshold cycle method was used for relative quantification of gene expression.

### Statistics

Prism V5.0d software (GraphPad, San Diego, CA) was used for statistical analysis. Unpaired t-test analyses were employed, and p-values were considered significant if less than 0.05. Bars on figures depict Standard Error of the Mean.

## Supporting Information

Figure S1 **HSC differentially express other markers that can be used to monitor HSC subsets in JAK2V617F mice.** (**A, B, D, E**) CD48^−^ LSK were gated and characterized with CD18, CD41, VCAM-1 and CD39 in the CD150^Hi^ fraction. (**C**) Altered ratios of CD86^−^ CD41^+^ and CD86^+^ CD41^−^ were found in the CD150^Hi^ HSC fraction. CD150^+^ CD48^−^ LSK were resolved with CD86 and CD41.
(PDF)

Figure S2 **Lineage associated transcripts in lymphoid and erythroid progenitors.** Real-Time PCR was performed using cDNA from sorted Flk2^+^ LSK and Lin^−^ ckit^Hi^ Sca1^−^ CD150^+^ CD105^+^ pre-CFUE. The data are representative of those obtained in two independent experiments.
(PDF)
