# Supplementary figures and images for: Stem and Progenitor Cell Subsets Are Affected by JAK2 Signaling and Can Be Monitored by Flow Cytometry
Source: PLoS One. 2014 Apr 3;9(4):e93643. doi: 10.1371/journal.pone.0093643 (PMC3974768; doi:10.1371/journal.pone.0093643)

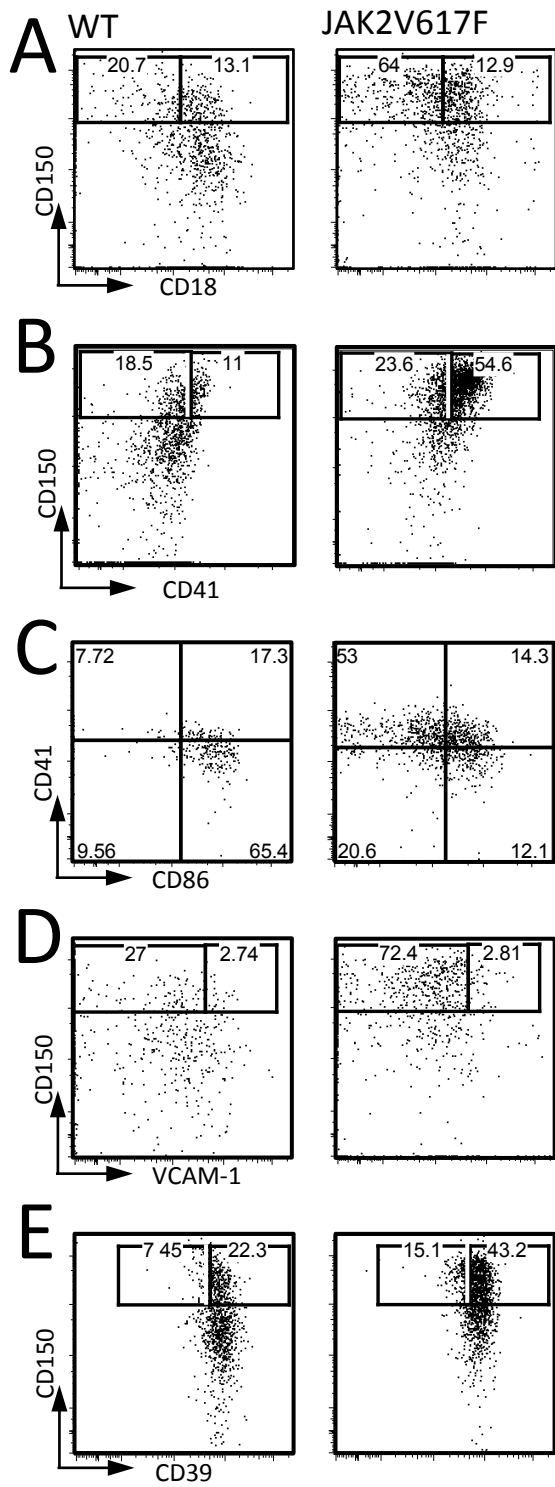

Figure S1. Iida et al

Supplement: Figure S1 — HSC differentially express other markers that can be used to monitor HSC subsets in JAK2V617F mice. (A, B, D, E) CD48− LSK were gated and characterized with CD18, CD41, VCAM-1 and CD39 in the CD150Hi fraction. (C) Altered ratios of CD86− CD41+ and CD86+ CD41− were found in the CD150Hi HSC fraction. CD150+ CD48− LSK were resolved with CD86 and CD41. (PDF) [file pone.0093643.s001.pdf]

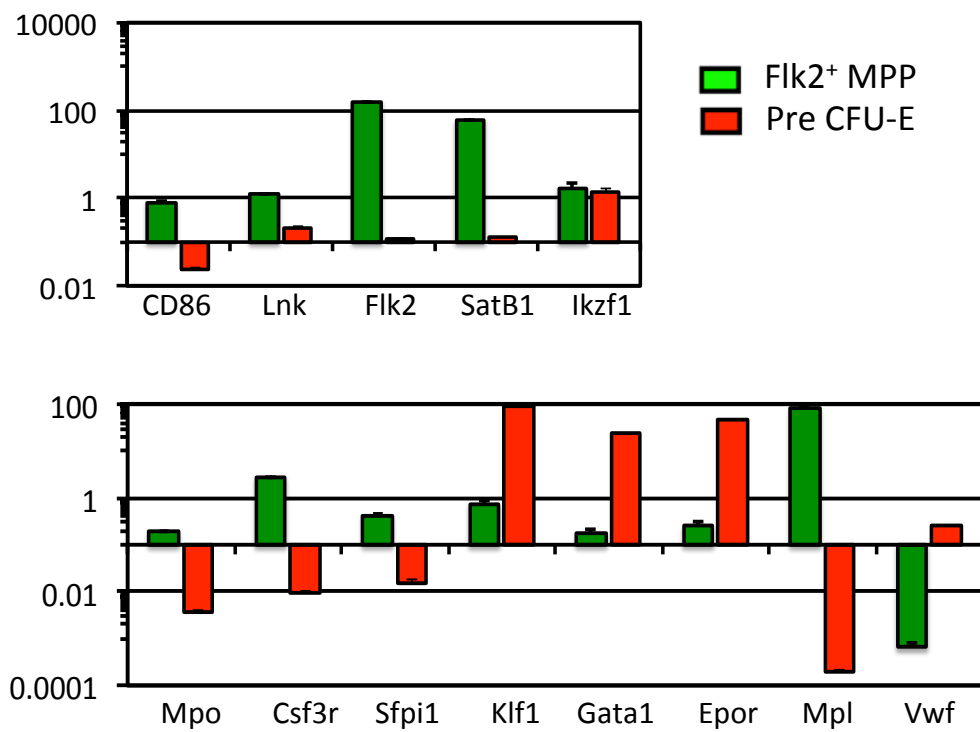

Figure S2. Iida et al

Supplement: Figure S2 — Lineage associated transcripts in lymphoid and erythroid progenitors. Real-Time PCR was performed using cDNA from sorted Flk2+ LSK and Lin− ckitHi Sca1− CD150+ CD105+ pre-CFUE. The data are representative of those obtained in two independent experiments. (PDF) [file pone.0093643.s002.pdf]
